# Anomalous origin of the left coronary artery from the pulmonary artery associated with an accessory atrioventricular pathway and managed successfully with surgical and interventional electrophysiological treatment: a case report

**DOI:** 10.1186/1752-1947-5-384

**Published:** 2011-08-16

**Authors:** Alexandros Tsoutsinos, Fotios Mitropoulos, Christina Trapali, John Papagiannis

**Affiliations:** 1 Department of Pediatric Cardiology, Onassis Cardiac Surgery Center, Syggrou Aven 356, Athens 176 74, Greece; 2 Pediatric and Congenital Heart Surgery, Onassis Cardiac Surgery Center, Syggrou Aven 356, Athens 176 74, Greece; 3 Department of Pediatric Cardiology, Aglaia Kyriakou Children's Hospital Thivon and Levadeias, Athens 11527, Greece; 4 Department of Pediatric Cardiology, Mitera Hospital, Erythrou Stavrou 6, Athens 15123, Greece

## Abstract

**Introduction:**

The combination of anomalous left coronary artery origin from the pulmonary artery and an accessory pathway has not been reported previously in the medical literature. In medicine, the coexistence of two clinical causes can lead to the same clinical findings, and this can make the researcher's attempt to distinguish between the two of them and, hence, the correct diagnosis and treatment difficult.

**Case presentation:**

A six-month-old boy from Pakistan was brought to our hospital with tachypnea and supraventricular tachycardia and, on the basis of echocardiography and multi-slice computed tomography, was diagnosed with an anomalous left coronary artery origin from the pulmonary artery. The presence of an anomalous left coronary artery origin from the pulmonary artery was not initially recognized, and left ventricular dysfunction was considered as a result of supraventricular tachycardia. He underwent direct re-implantation of the left coronary artery to the aorta using the trapdoor flap technique. Recurrent episodes of supraventricular tachycardia resistant to maximal pharmacological treatment occurred post-operatively. A left posterolateral accessory pathway was successfully ablated by using a trans-septal approach.

**Conclusions:**

It should not be forgotten by anyone that many times in medicine what seems obvious is not correct. It can be difficult to distinguish two clinical entities, and frequently one is considered a result of the other. This is the first report of the coexistence of an anomalous left coronary artery origin from the pulmonary artery and recurrent supraventricular tachycardia due to an accessory pathway in a child that was treated successfully with combined surgical and interventional electrophysiological treatment. This case may represent a first educational step in the field of congenital heart disease, that is, that anomalies such as an anomalous left coronary artery origin from the pulmonary artery may be concealed in a child with other serious cardiac problems, in this case mitral regurgitation, dilation of the left ventricle, and recurrent episodes of tachycardia.

## Introduction

Anomalous left coronary artery origin from the pulmonary artery (ALCAPA) is a rare congenital cardiac malformation requiring surgical treatment in infancy. To the best of our knowledge the combination of ALCAPA and an accessory pathway and its treatment with radiofrequency catheter ablation (RFCA) has not been described previously.

### Case presentation

**Figure 1:**
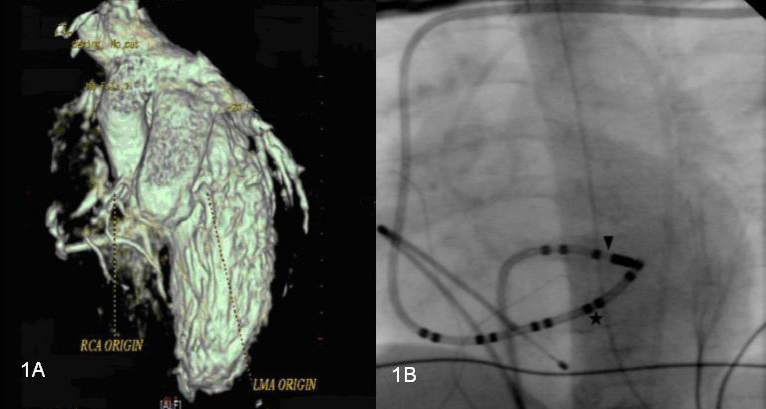
**(A) Multi-slice computed tomographic image of the anomalous origin of the left main coronary artery from the pulmonary artery, and (B) location of the successful ablation site at the left posterolateral area**. Shown are the coronary sinus catheter (*) and the ablation catheter (inverted filled triangle).

**Figure 2:**
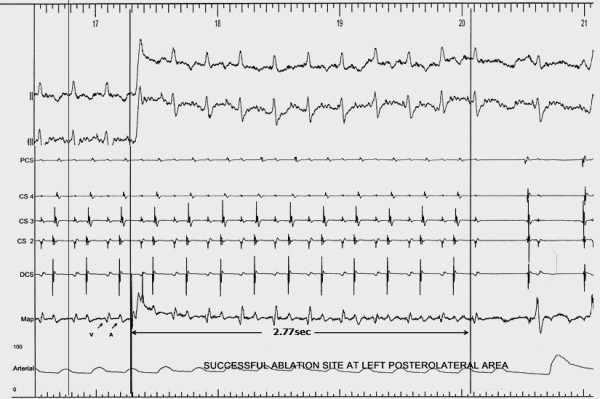
**Interruption of tachycardia during radiofrequency current application at the left posterolateral area**.

A six-month-old boy (weight 6.3 kg) presented to our hospital with episodes of supraventricular tachycardia (SVT), tachypnea, and left ventricular dysfunction. The presence of ALCAPA was not initially recognized, and our patient's left ventricular dysfunction was attributed to SVT. He was eventually diagnosed with ALCAPA on the basis of echocardiography and multi-slice computed tomography (CT) (Figure 1A). Τhe suspicion of probable ALCAPA was raised after his third echocardiographic examination and was confirmed by a CT scan. The left coronary artery originated from the leftward-facing sinus of the pulmonary valve. The left ventricle was dilated with an ejection fraction of 30%. Our patient underwent direct re-implantation of the left coronary artery to the aorta using the trapdoor flap technique (cross-clamp time 92 minutes, bypass time 137 minutes). He started having recurrent episodes of SVT, with a heart rate of 220 beats/minute immediately after extubation on the second post-operative day. The episodes were converted to sinus rhythm with adenosine or rapid atrial pacing, thus ruling out junctional ectopic tachycardia. Despite treatment with amiodarone, the episodes continued. Propranolol, digoxin, and propafenone were added at maximal tolerated doses without success. An electrophysiological study was performed in the fourth post-operative week using a 5-French decapolar catheter placed into the left subclavian vein in the coronary sinus (CS), a 4-French bipolar catheter placed from the left femoral vein into the right ventricle, and a 5-French mapping/ablation catheter placed through the right femoral vein. Atrioventricular re-entry tachycardia was induced reproducibly with programmed atrial stimulation, with a tachycardia cycle length of 250 milliseconds and earlier retrograde atrial depolarization recorded by the distal bipole of the CS catheter. Access to the left atrium was achieved by using a trans-septal approach (Figure 1B), and mapping was performed during tachycardia using a non-fluoroscopic navigation system (Ensite-NavX; St Jude Medical, St Paul, MN, USA). Tachycardia stopped 2.7 seconds after the onset of the fourth application of RF energy (Figure 2). The total fluoroscopy time was 24.7 minutes, maximum power was 30W, maximum temperature was 58°C, and the total procedure duration was four hours. Post-ablation aortography revealed patency of the left coronary artery without stenosis of the circumflex coronary artery. There was no recurrence of the SVT while our patient was in a drug-free state at the six-month follow-up examination.

## Discussion

ALCAPA is a rare congenital cardiac malformation in infancy (1 in 300,000 live births) [1] that produces a coronary steal phenomenon and usually requires surgical treatment in infancy. It appears with features of myocardial ischemia or cardiac failure and may be mistaken for dilated cardiomyopathy [1]. During the fetal period this anomaly probably has no harmful effects, as the oxygen pressure and saturation levels are similar in the aorta and pulmonary artery. Myocardial perfusion is presumably normal. After birth, however, the pulmonary artery contains desaturated blood at pressures that fall below systemic pressures. The left ventricle is perfused with desaturated blood at low pressures. The collateral flow is initially low. At first, ischemia is transient and occurs only with exertion, such as feeding or crying, but further increases in myocardial oxygen demand lead to infarction of the anterolateral left ventricular free wall, with resultant compromise of left ventricular function. This causes congestive heart failure, which is often made worse by mitral regurgitation secondary to a dilated mitral valve ring or infarction and dysfunction of the anterolateral papillary muscle.

The surgical treatment initially described in the literature was ligation of the left coronary artery [1]. Since then, several other surgical approaches have been described [1,2], such as subclavian-to-left coronary artery anastomosis, direct re-implantation of the anomalous artery to the aorta, or Takeuchi repair (with an intra-pulmonary baffle). Currently, re-establishment of the dual coronary system is considered the best approach [2].

Tachycardia-induced cardiomyopathy, another aspect of the malformation, is a form of dilated cardiomyopathy and heart failure caused by supraventricular and ventricular tachyarrhythmias. The clinical manifestations of heart failure are associated with ventricular systolic dysfunction and dilation associated with persistent tachyarrhythmias. The condition is generally considered to be reversible, with normalization of heart rate. In our patient, the initial cause was not immediately obvious. The occurrence of SVT in infancy is well known, but to the best of our knowledge the combination of ALCAPA and SVT in babies and children requiring treatment with catheter ablation has not been described previously. The most common cause of SVT in babies is an accessory pathway. Although RF ablation has become the treatment of choice in older children with recurrent SVT [3-6], the application of this treatment in infancy is undertaken only after failure of pharmacological therapy and in patients with life-threatening arrhythmias. The main reasons for this approach are the natural history of SVT with resolution of episodes in infancy [3-6], the risk of damage to the coronary arteries and intra-cardiac structures, and technical reasons (patient and catheter size and curves) [7]. A large multi-center study by the Pediatric Electrophysiology Society as well as other reports have shown that, when performed by experienced operators, RF ablation in babies has similar success and complication rates to those in older children [7,8]. Complications may occur, however, and appear to be related to structural abnormalities of the heart (which are significantly more common in babies), the size of the child [7,8], and the total number of lesions. Several reports have mentioned injury of the coronary arteries in small children after RFCA. We were particularly concerned about damage to the coronary circulation in our patient, especially after re-implantation of the left coronary artery, and for this reason we were very cautious during RF lesion treatment. Another approach that may be considered is cryoablation, which creates smaller and shallower lesions. The disadvantages of this method are the larger size of the catheter and its stiffness. We elected to use RF energy and minimized the number of catheters used and the number, power, and duration of the lesions. By using this approach, safe and successful ablation of the accessory pathway was achieved.

Other congenital anatomic defects that manifest in infancy like SVT are Ebstein anomaly and levotransposition of the great vessels, atrial isomerism, hypertrophic obstructive cardiomyopathy, Uhl's anatomy, and arrhythmogenic right ventricular dysplasia.

There are two theories regarding the development of the ALCAPA anomaly: the older embryological theory of Abrikossoff abnormal septation of conotruncus into the aorta and pulmonary artery, and the newer theory of Hackensellner. Hackensellner's theory can explain all known and possible varieties of anomalous coronary arteries. In brief, all six semi-lunar valve regions of the aorta and pulmonary artery have the propensity to develop anlagen of the coronary arteries. The various anomalies are explained on the basis of faulty involution or persistence of one or several of these anlagen. From the other side, by definition, accessory atrioventricular pathways are aberrant muscle bundles that connect the atrium to a ventricle outside the regular atrioventricular conduction system [9]. In the embryonic human heart, a ring of musculature at the atrioventricular canal provides myocardial continuity between the developing atrial and ventricular myocardium in the early stages. The canal myocardium is sandwiched between sulcus tissue on the outside and endocardial cushions on the inside. Wessel *et al. *suggested that accessory pathways result from incomplete fusion between sulcus and cushion tissues. In contrast, a simpler explanation has been put forward by Ho [9], who suggested that invagination of sulcus tissue, such as a wedge through the muscular canal wall, is part of the process of the development of valvular leaflets, with little contribution from the cushions.

Gittenberger-de Groot *et al*., while studying the embryologic origins of the coronary vessels in chicken-quail chimeras, identified the migration of a novel population of cells termed 'epicardial-derived cells' (EPDCs) into the myocardial interstitium and endocardial cushions. Observing a close relationship between EPDCs and cardiac fibroblasts, they suggested a potential role of migrating EPDCs in the formation of the insulating tissue plane between atrial and ventricular myocardium. Developmentally, the work of Kolditz *et al. *would appear to support the notion that accessory pathways result from incomplete interruption of canal myocardium due to the late arrival of EPDCs.

Therefore, we theorize that a probable connection of the theories regarding the genesis of the ALCAPA malformation and the creation of an abnormal atrioventricular tissue connection, as mentioned above, is perhaps responsible for the simultaneous combination ALCAPA and SVT.

## Conclusions

The discrimination and diagnosis of two illnesses that develop simultaneously is difficult, and often one is considered a consequence of the other. It should not be forgotten that, in medicine, the coexistence of two clinical entities can lead to the same clinical result and also that the first obvious diagnosis (in our patient, SVT) can hinder the detection of an essential underlying clinical entity. To the best of our knowledge, this is the first case report on the coexistence of ALCAPA and recurrent SVT in infancy due to an accessory pathway that was treated with successful combined surgery and interventional electrophysiology.

## Consent

Written informed consent was obtained from the patient's next-of-kin for publication of this case report and any accompanying images. A copy of the written consent is available for review by the Editor-in-Chief of this journal.

## Competing interests

The authors declare that they have no competing interests.

## Authors' contributions

TA analyzed and interpreted the data from our patient and participated in the EP study. MF was the cardiac surgeon who performed the operation and implanted the LCA into the aortic annulus. TC suspected and diagnosed ALCAPA. PJ performed the EP study. All authors read and approved the final manuscript.
